# Practical Notes on Diagnosis and Treatment

**Published:** 1910-12-17

**Authors:** 


### Practical Notes on Diagnosis and Treatment.

**Breast Plasters.**

A belladonna plaster is probably no better than any ordinary plaster for the purpose of allaying the secretion of milk.—*Professor Dixon.*

**Fracture of the Skull.**

Hæmorrhage from the external auditory meatus should never be interfered with by plugging, and syringing the meatus is absolutely contraindicated.—*Mr. T. H. Kellock.*

**Tuberculous Glands.**

In all cases tuberculin treatment should be continued for some months after apparent recovery, in order to diminish the tendency to relapse. In many cases auto-inoculation is a valuable adjuvant; better results are often obtained by inducing auto-inoculation by means of a stimulating dose of $x$-rays, or by means of a hot sand-bag.—*Dr. Arthur Latham.*

**Ovaritis.**

The ovaritis of the text-books is a most misleading disease, and belongs to a past age in gynæcology. True inflammation of the ovary is unknown apart from infection, and the commonest source of infection is *viâ* the Fallopian tubes. Hence ovaritis is unknown apart from salpingitis, with the exception of certain puerperal infections, in which the ovary becomes inflammed by an infection following the lymphatics, and generally ends in acute abscess formation.—*Dr. T. G. Stevens.*

**Trauma and Malignant Tumours.**

The subject of trauma as a factor in producing cancer has assumed importance in consequence of cases brought into the courts in connection with the Workmen's Compensation Act. Trauma has only seriously been advanced in the case of the breast. It may be useful to know that though the majority of women do give a history of some antecedent injury in cases of carcinoma mammæ, yet it is probably true that not one in a thousand of women who have received a blow on the breast becomes the victim of cancer.—*Mr. Bland Sutton.*

**Lime in the Eye.**

Lime is apt to produce a considerable degree of destruction, but, fortunately, it is more or less slaked. A strong solution of cane sugar should be used to wash the eye out, not water, as it would help to increase the slaking. Atropine ointment and a cold-water compress may be then applied.—*Dr. J. Cunningham.*

**Asthma.**

There are many drugs which will check the paroxysm. Among the most useful is caffein given by the mouth or by subcutaneous injection, or in the form of black coffee. Another useful drug is nitrite of amyl; this produces great vascular dilatation, and may give immediate relief. Iodide of ethyl often does good; it is a volatile substance and contains a large amount of iodine in a readily absorbable form. Pilocarpin sometimes acts when others fail, and adrenalin, which has an exactly opposite action, is also often very effective.—*Dr. Samuel West.*

**Cyclical Albuminuria in Children.**

This affection, in which albumin is present in the urine whilst the patient is up and about, but disappears after rest in bed, may be regarded as one of the neuroses of childhood. It occurs most commonly in children at the approach of puberty, but may be met with as early as the eighth year. The subjects are nervous, emotional, irritable, or passionate. They are peculiarly liable to night terrors, migraine, or abdominal pain. Vasomotor disturbances are common, and the children are often the subjects of so-called "mucous disease." There is no evidence that the albuminuria is due to nephritis of any sort, nor that the condition ever leads to granular kidney or to any form of Bright's disease. Treatment by drugs is unsatisfactory, but in some cases calcium chloride has seemed to be of use. General principles can only be followed, with a regular course of life, freedom from emotional excitement, and overwork; above all, the subjects must not be treated as invalids.—*Dr. Leonard Guthrie.*

